# Variation in sexual signals and defensive strategies elicits receiver-dependent shifts in attractiveness

**DOI:** 10.1242/jeb.250360

**Published:** 2025-07-25

**Authors:** Brian C. Leavell, Dineilys Aparicio, Hoover Pantoja-Sánchez, Rachel A. Page, Ximena E. Bernal

**Affiliations:** ^1^ Department of Biological Sciences, Purdue University, West Lafayette, IN 47907, USA; ^2^ Smithsonian Tropical Research Institute, Balboa, Ancón, Apartado Postal 0843-03092, Panamá

**Keywords:** Female choice, Foraging, Inducible defense, Predator–prey interactions, Vibratory cues, Eavesdropper, *Engystomops pustulosus*

## Abstract

Sexual selection often favors the evolution of conspicuous mating displays. Emitting such overt displays carries the risk of interception by eavesdropping enemies, i.e. predators, parasitoids and parasites that exploit communication systems to find and attack their signaling victims. Yet, many signalers respond to variation in perceived eavesdropper risk, protecting themselves through risk-dependent inducible defenses to mitigate potential costs. Given that signalers are embedded in communication networks in which they interact with other signalers, target receivers and multiple eavesdropping enemies, here we investigated how variation in signaling and defensive strategies impacted by an eavesdropping enemy (frog-biting midges; Diptera: Corethrellidae) affects other receivers in a communication network. Ultimately, we aimed to determine whether and to what extent effects that cascade throughout the network shape relative fitness among chorusing males. Using female choice experiments with túngara frogs (*Engystomops pustulosus*) and predation experiments with eavesdropping, fringe-lipped bats (*Trachops cirrhosus*), we show that variation in the call elaboration and defensive strategies of competing males shapes their relative fitness. Defensive strategies targeting eavesdropping frog-biting midges indirectly shift a male's relative attractiveness to females and predatory bats, though the mechanisms and impacts are context and receiver specific. These findings showcase how the frequency-dependent effects of micropredation can dynamically shape variation in secondary sexual characteristics and thus influence the mechanisms driving sexual selection.

## Teaser

**Summary:** The degree to which calling male túngara frogs defend themselves from eavesdropping frog-biting midges alters their attractiveness to females and predatory bats, though the sensory mechanisms and effects differ per receiver.

## INTRODUCTION

Prey face the challenge of obtaining resources while under threat of attack from multiple natural enemies, including predators, parasites and parasitoids (Baker and Smith, 1997; Sih et al., 1998; Decaestecker et al., 2002). Signaling prey are particularly exposed as they often generate conspicuous stimuli that propagate throughout the environment and are potentially detectable to a community of receivers. In communication networks, signalers target particular receivers, but their signal can be intercepted by non-target receivers such as eavesdropping natural enemies who exploit the signal to identify and localize their prey. To preclude attacks, signalers can combat these eavesdroppers with signaling strategies that limit the probability of being detected and localized (Belwood and Morris, 1987; Cummings et al., 2003; Wilson and Hare, 2004; Ruxton, 2009; Searcy and Yasukawa, 2016; Legett et al., 2019; Bernal and Page, 2023). However, constraints imposed by many factors, including the environment (Endler, 1992; Gomez and Théry, 2004) and eavesdropper sensory adaptations (Zuk and Kolluru, 1998), may prohibit the use of these strategies. Instead, upon attack, inducible physical defenses such as aggressive behaviors (e.g. Bateman and Fleming, 2013) might be used by signalers to thwart attacks (Harvell, 1990).

Given the general conspicuousness of signals, especially highly ornamented signals used to attract mates, shifts in a signaler's behavior that result from defensive strategies are potentially salient to a suite of receivers. Yet, it largely remains an open question whether a signaler's behavioral modifications directed to temporarily reduce risk from one type of eavesdropping enemy can dynamically shape how target and non-target receivers in the network perceive and respond to the signaler (for an exception, see Legett et al., 2019). In non-communication predator–prey contexts, defenses that target specific enemies are predicted to increase the risk of attack from other predators (Matsuda et al., 1996). However, multiple predator effects may be difficult to generalize, as they can reduce or increase predation risk, or even have no effects, depending on the context (Sih et al., 1998).

Uncovering how multiple receivers influence each other carries relevant implications for our understanding of the selective forces on signal design and the evolution of communication systems. Here, we asked how one eavesdropping enemy modulates the behaviors of other receivers in the communication network. We addressed this question by examining the responses of target and non-target receivers associated with the same signaler. Specifically, this study investigated the impact of eavesdropping frog-biting midges (Diptera: Corethrellidae), via the ‘swatting’ defense they elicit in calling male túngara frogs (*Engystomops pustulosus*), on the mating decisions of female túngara frogs and foraging decisions of an eavesdropping predator, the fringe-lipped bat (*Trachops cirrhosus*).

Frog-biting midges (∼1–2 mm wing length; Borkent, 2017) detect and localize their frog hosts by listening to the mating calls of frogs (McKeever, 1977; Bernal and de Silva, 2015). In just 30 min of calling, males can suffer attacks from hundreds of midges (Bernal et al., 2006), though attack frequency varies widely. To combat these attacks, túngara frogs swat at blood-sucking midges with their arms and legs (McKeever, 1977; Bernal et al., 2006; Leavell et al., 2022). The more a signaler is attacked by midges, the more likely he is to swat. Doing so, however, indirectly limits his call rate and complexity via a temporal trade-off between swatting and calling (Leavell et al., 2022). Variation in call rate, complexity and swat rate appears to be, in part, driven by variation in midge attack frequency (Leavell et al., 2022). Given that call rate and complexity are positively correlated with attractiveness to both females and eavesdropping bats (Tuttle and Ryan, 1981; Akre et al., 2011; Ryan et al., 2019), it is possible that midges shift the fitness landscape of a chorus via differential suppression of call elaboration among competing males.

Defensive swats, however, also create ripples in the water that are similar to the ripples generated by túngara frogs as a byproduct of their mating call (Leavell et al., 2023). Female túngara frogs appear to integrate call-induced ripples when discriminating among mating signals (Cronin et al., 2019; James et al., 2022), as do fringe-lipped bats, which seem to prefer calls with ripples (Halfwerk et al., 2014). Swat-induced ripples often occur in close temporal proximity to calls and are greater than call ripples in intensity and frequency (Leavell et al., 2023) – features predicted to increase their saliency to both target and non-target receivers. As in a typical signal detection dilemma, similarities among stimuli of different underlying conditions force receivers to make appropriate decisions in response to ambiguous information (Leavell and Bernal, 2019). Male frogs appear to generalize the meaning of both call- and swat-induced ripples of male competitors, responding to each by increasing their call rate and complexity (Leavell et al., 2023). How female túngara frogs and frog-eating bats respond to swat-induced ripples is unknown.

As with male competitors, it is possible that both females and bats may also generalize the meaning of call- and swat-induced ripples, showing greater attraction to males that generate more swat ripples. Yet, the ‘just-meaningful difference’ (*sensu* Nelson, 1988) in a stimulus characteristic – i.e. the degree to which a stimulus must change to result in a significant change in a receiver's response – can differ between female túngara frogs and eavesdropping bats. For example, generally a male that swats at midge attacks reduces his call elaboration, while the ripples generated by his swats cause his neighboring rival's call elaboration to increase. When solely considering the difference in call elaboration between two such males, the effect of the midges shifts female preference towards the prey's rival and away from the swatting prey, while having no impact on the foraging preferences of eavesdropping bats (Leavell et al., 2023). Considering that both females and fringe-lipped bats integrate stimuli across sensory modalities, it remains to be seen whether the presence of swat ripples affects the decision making of either receiver.

To unravel how variation in calling and defensive behaviors translates into relative fitness of competing strategies, we experimentally assessed the relative fitness of signaling males that vary across these behavioral dimensions. We tested the hypothesis that midges modulate mating opportunities and predation risk of competing males by altering both their calling and defensive strategies. We predicted that the magnitude of a male's call rate, call complexity and swat rate, and their interactions, will be positively associated with female and bat preference.

## MATERIALS AND METHODS

### Study site

This research was performed at the Smithsonian Tropical Research Institute (STRI; Gamboa, Panamá; 9°07.0′N, 79°41.9′W). Frogs and bats were collected and tested within 2 km of STRI facilities.

### Experimental stimuli

To represent the variation in calling behaviors from swatting frogs, we created ripple and airborne treatment playback stimuli based on previously recorded samples from our study population (described in Leavell et al., 2022, 2023). The range of calls used captures natural variation in male calling behavior under diverse micropredator densities and intensities of male–male competition. Following Ryan and Rand (2003), we *z*-transformed the values for call rates, swat rates and total chucks (all recorded over 50 continuous calls per male). We then used Euclidian distances to select the samples that represented the range of trait space: the sample closest to the mean (i.e. *x*=0, *y*=0, *z*=0; where *x*=call rate, *y*=swat rate, *z*=total chucks), eight samples at approximately ±1 s.d. and eight samples at extreme values (Figs S1 and S2). The experimental design was tailored to increase statistical power given the distinct ecological contexts in which bats and female frogs make decisions. Because frog-eating bats consume about half their body weight per night (∼15 g) and readily participate in many foraging trials per night, they can be tested multiple times over the course of a night (Hemingway et al., 2020). Thus, all 17 playback files were used in the bat foraging experiment (43.0±13.9 trials per bat, mean±s.d.; *n*=5 bats). Conversely, female túngara frogs typically respond to fewer mate choice trials before losing their motivation to perform phonotaxis (5.2±2.6 trials per female; *n*=32 females). To maximize the range of male traits played back to females, while minimizing the experimental trials presented to them, we therefore focused on the eight extreme samples in the female choice experiment.

Each experiment was a two-choice preference test in response to simultaneous playback of airborne calls with call-induced ripples and swat-induced ripples. We constructed 1 min audio files in Adobe Audition that captured the call rate and average complexity for these samples, as well as audio files that captured the rate of ripple production based on the sample's swat rate. For samples that featured many ornamental ‘chucks’, we appended either one or two chucks to calls in a randomized order to reach the appropriate number of chucks for our 1 min playback file. Audio files were looped for experiments. In a trial, a treatment playback from the multivariate dataset (consisting of airborne calls with call-induced ripples and swat-induced ripples) was played concurrently with an average call from the population [i.e. 0.5 calls s^−1^ and 1 chuck call^−1^ (Ryan, 1985), with accompanying call ripples, but no swat ripples]. Swat audio files (i.e. audio of swat-induced ripple vibrations) were always played back between calls. For one swat, the middle of the swat audio file was positioned at the middle point between the end of the first call and the start of the second. For two swats, the middle of the first swat was positioned at the middle point, as was the start of the second swat. For three swats, the end of the first swat, the middle of the second swat and the start of the third swat occurred at the middle point of the calls.

### Female choice

In August 2022, we collected mated (i.e. in amplexus with a male) female túngara frogs, *Engystomops pustulosus* (Cope 1864) (*n*=32), after sundown, between 19:30 h and 01:00 h. This research was conducted in accordance with STRI institutional animal care and use protocol SI-22042 and Panamanian legal and ethical regulations (Ministerio de Ambiente permit ARG-097-2022). Following protocols established by the American Society of Ichthyologists and Herpetologists (https://asih.org/animal-care-guidelines) and previous work on this species and other amphibians (Ryan, 1985; Kinkead et al., 2006; Grafe et al., 2011), we toe-clipped all frogs to prevent pseudoreplication and released each frog with its mate on the same night and at the individual's point of capture. We conducted phonotaxis experiments in a dark, acoustically isolated chamber (2.8 m×2.8 m×2.8 m; 25.0–25.7°C). Inside the chamber was a wood-framed, plastic-lined pool (2.23 m×0.60 m×0.07 m) filled with dechlorinated tap water to a depth of 1.5 cm. To produce airborne playback, two small speakers (Fostex FE103En) in custom-built cabinets were positioned ∼1 m across from and facing each other. All speakers were amplified through Pyle PTA4 amplifiers. The airborne playback speakers sat slightly above the water, supported by 2 cm PVC ‘tee’ fittings that rested in the water. The mid-point between the speakers, which was also the mid-point of the chamber's opposing walls, was the center of the arena and the female's entry point at the beginning of each trial.

To produce ripples, we played back audio of ripple waveforms previously sampled with a laser vibrometer (described in Leavell et al., 2023) through custom-built loudspeakers (as described in Halfwerk et al., 2016) placed outside the arena. A vinyl tube was fitted to the opening of each loudspeaker on one end and ran along a PVC frame (97 cm×81 cm×33 cm) to which it was secured. At the other end of the tube was a nozzle that rested adjacent to its associated airborne speaker and perpendicular to the water surface such that a small meniscus formed around the nozzle. Following the methods in Leavell et al. (2023), we calibrated call and swat ripple playbacks before and after experiments using the digital output of a digital laser vibrometer (Polytec PDV-100; velocity 20 mm s^−1^, low pass 22 kHz, high pass none), focused on a reflective marker floating on the water surface. Calibrations were recorded using a Marantz audio recorder (PMD661 MKII; 48 kHz sample rate, 24 bit). All calibration recordings were subsequently processed using custom code to derive velocity and frequency measurements. Though there was some variation across playbacks, the vast majority fell within the natural range of call and swat ripple measurements described in Leavell et al. (2023) (see R script: https://doi.org/10.5281/zenodo.14860389): maximum velocity (mm s^−1^ peak to peak): range ∼2–10 (call), ∼1.25–10 (swat); median ∼3.5 (call), ∼6.0 (swat); dominant frequency (Hz): range ∼3–12 (call), ∼3–16 (swat ripple); median ∼5.8 (call), ∼8.0 (swat).

To observe female behavior, we illuminated the room with infrared lights (Sima Model SL-100IR) and recorded video with a Sony Handycam (FDR-AX33) in night-shot mode. At the start of a trial, we broadcast the simultaneous playback of male stimuli. Females were allowed to emerge from the end of a PVC tube at the center of the arena (see Movie 1 for example trial). We considered a choice was made when the female paused within 10 cm of a speaker (front or back) or moved without stopping within one body-length of the nozzle. See Supplementary Materials and Methods for further details. We interpreted female preference as a proxy for relative male fitness. We also measured the time it took from emergence into the arena until a female made a choice, which we refer to as ‘latency to choose’. Before and after testing a female, we ensured that the maximum intensity of the whine call component was set to 82 dB sound pressure level (SPL; peak, C weighting, fast; re. 20 µPa) at the female's point of release. This intensity matches natural call intensities at 1.0 m from a calling male. SPL was measured with a Brüel & Kjær 2238 Mediator Sound Level Meter (peak, C weighting, fast).

### Bat foraging

From January to March 2019, we captured and tested wild-caught *Trachops cirrhosus* (Spix 1823) within Soberanía National Park, Panamá. To ensure no individual was re-used in experiments, each bat was marked with a passive integrated transponder (PIT tag, 12 mm, ∼0.1 g and ∼0.3% of body mass; Biomark, Boise, ID, USA). Following testing, all bats were released at their point of capture. This research was conducted in accordance with a STRI institutional animal care and use protocol 2017-0102-2020-A8 and Panamanian legal and ethical regulations (Ministerio de Ambiente permit SE/AP-13-18).

We sourced the stimuli and calibrated the playback system as described in the female choice experiments. Given that these bats hunt from above, speakers were placed ∼1 m apart, facing up, over the center of neighboring pools of water in an outdoor flight cage (5×5×2.5 m). Water depth was maintained at ∼3.0 cm. To present the multimodal stimuli as though they were coming from a single male, the ripple speaker and vinyl tube setup used in the female choice experiment was arranged such that the nozzle was nearly under the speaker. Following previous studies with this bat species (Halfwerk et al., 2014), we first trained bats (*n*=5) to hunt at two small feeding platforms (i.e. acoustically transparent mesh atop the speakers' diaphragms). As in the experiments with female frogs, speakers were positioned 1 m from each other over the pools of water. For training, we used a mix of the playback stimuli as well as recordings of the mating calls of other local frog species. Bats learned to begin foraging from a corner roost positioned equidistant (4 m) from each speaker. Once the bats were trained, we began the experiment.

This species approaches unrewarded speakers broadcasting frog calls and is not attracted to food rewards per se (Page and Ryan, 2008). However, across repeated trials, individuals can lose motivation if not rewarded or appear to choose random speakers if constantly rewarded (B.C.L., personal observation). To motivate bats to repeatedly perform the foraging preference task, we presented a food reward (fish) on both speakers at a 50% reward rate, such that in 50% of trials both speakers were baited and in 50% neither were baited (randomized across trials, with no more than four sequential rewarded trials). To control for potential use of echolocation or chemosensory cues across trials, we used size- and shape-matched stones covered with fish ‘juice’ when the speaker pair was not rewarded.

We recorded video of bat trials using a recording setup similar to the female choice experiment (see Movie 2). From the video recordings, we measured the bat's choice, latency to respond (time at which bat first takes flight−playback start time) and flight time (time a choice was made−time at which bat first takes flight). All trials in which a bat did not respond were coded as N/A. When a bat landed on one speaker and then flew again and landed on the other, we coded the time at which the bat landed on the first speaker.

### Statistical analysis

All statistical analyses were performed in R version 4.0.3 (http://www.R-project.org/). For our analyses, we formulated Bayesian regression models using the *brms* package version 2.18.0 (Bürkner, 2017). For female and bat preference experiments, we fitted mixed logistic regression models of choice between treatment and control playbacks, with call rate, total chucks and swat rate as *z*-transformed fixed effects and the individual receiver as a random intercept. We then examined the individual and interactive effects of call rate, swat rate and total chucks on mate choice in female túngara frogs and prey choice in predatory bats. For further inference of female behavior, we fitted generalized linear mixed models of female latency to choose a male. We compared models with log-normal, shifted log-normal, Poisson and negative binomial error structures. All latency models had the same fixed and random effects as described above. Models were compared by assessing expected log pointwise predictive densities with the loo package version 2.4.1 (Vehtari et al., 2017).

For all models we used weakly informative priors to ensure that draws from the prior could be from any hypothetical dataset (Gabry et al., 2019). In this way, these priors include probability mass around potential, but not implausible extreme values. We ran 4 chains with 3000 iterations each and discarded the first 500, resulting in 10,000 posterior samples. To ensure model convergence, we visually examined traceplots for good mixing of chains and confirmed that 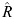 <1.1 for all parameters. We performed posterior predictive checks to assess model fit with the bayesplot package version 1.8.1 (https://CRAN.R-project.org/package=bayesplot). Strength of evidence for effects was determined following the framework established in Muff et al. (2022) and Kruschke and Liddell (2018), using posterior probability distributions degree of overlap with values of interest. One-sided tests using the hypothesis function in *brms* were used to ascertain the strength of evidence for posterior probability distributions of beta parameters having positive or negative effects, where the range 0.00–0.84 indicates little or no evidence, 0.85–0.89 is weak evidence, 0.90–0.94 is moderate evidence, and 0.95–1.00 is strong evidence. See https://doi.org/10.5281/zenodo.14860389 for details specific to each model.

## RESULTS

**Fig. 1.**
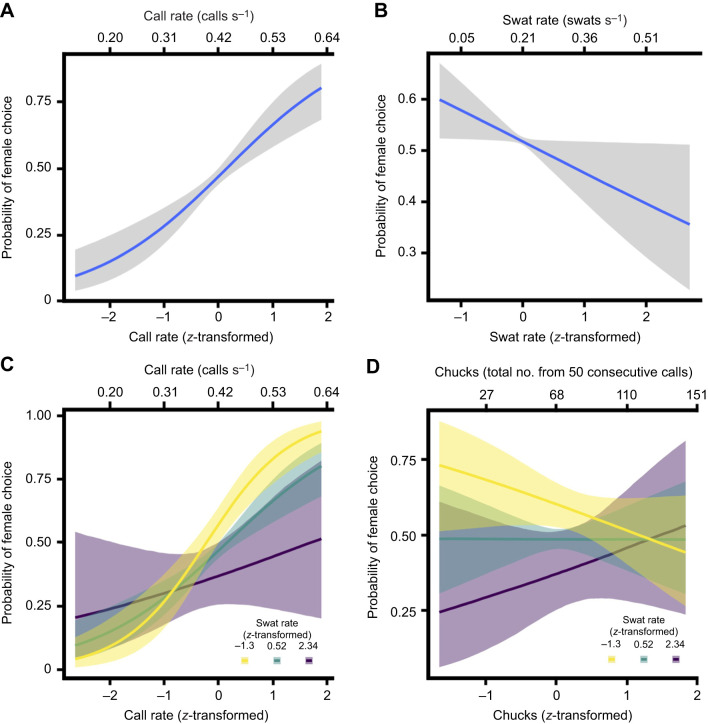
**The effects of male túngara frog call and swat rates on the probability of females selecting treatment (1) or control (0).** Effects of call rate (A), swat rate (B) and their interaction (C) on female choice, and the effects of the interaction between a male's call complexity (i.e. number of chucks) and swat rate (D). All other parameters in the model were held at mean values to derive these functions. The solid lines and shading indicate median estimates and their associated 90% credible intervals.

Faster-calling males were more likely to attract females, with the odds increasing 2.6 times [95% credible interval (CI) 1.79–4.17, Prob(β>0)=1; Fig. 1A] with one standard deviation increase in call rate. There was also strong evidence that female preference was impacted by male swat rates [Prob(β<0)=0.95]. Females were 0.79 times (95% CI 0.60–1.05; Fig. 1B) less likely to choose a male for every 1 s.d. increase in swat rate. Moreover, there was also strong evidence that the interaction between call and swat rates influences female choice [Prob(β<0)=0.96, odds ratio=0.76, 95% CI 0.54–1.04; Fig. 1C], such that females were less likely to prefer fast-calling males the more often they swatted. There was moderate support for the interaction between a male's call complexity (no. of chucks) and swat rate having an effect on female choice [Prob(β>0)=0.90, odds ratio=1.20, 95% CI 0.91–1.60; Fig. 1D], such that females were potentially more likely to choose males with less complex calls if they swatted less often. We found no support for female choice being influenced by the number of chucks alone (odds ratio=0.91, 95% CI 0.58–1.45), the interaction of call rate and chucks (odds ratio=0.88, 95% CI 0.48–1.57), or the interaction of call rate, chucks and swat rate (odds ratio=1.15, 95% CI 0.69–1.93).

**Fig. 2.**
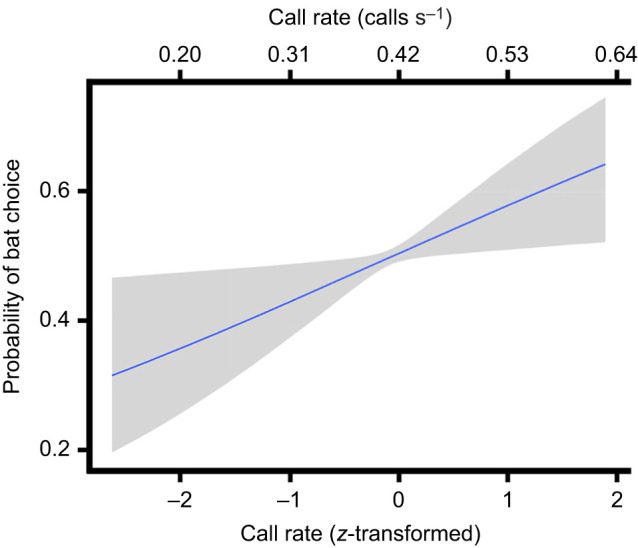
**Probability of fringe-lipped bat selecting treatment (1) or control (0).** For this estimate, swat rate and total chucks were held at mean values. The solid lines and shading indicate median estimates and their associated 90% credible intervals.

Female latency to choose a mate was explained by neither call nor swat characteristics, nor any of their interactions (see R script output for comprehensive β estimates: https://doi.org/10.5281/zenodo.14860389). The same was largely true for bat foraging behavior. A frog's call rate, however, influenced bat preference. The odds of a bat preferring the treatment playback over the control increased 1.40 times (95% CI 1.02–1.93; Fig. 2) for every 1 s.d. increase in call rate.

## DISCUSSION

In this study, we asked how one eavesdropping enemy impacts the decision making of target and other non-target receivers in a communication network. We found that eavesdropping frog-biting midges influence other receivers in multiple ways. Water ripple stimuli elicited by anti-midge swats impact female mating decisions such that females are less likely to mate with males that more frequently swat at attacking midges. While we predicted that females would respond to swat ripples, the relationship between swat rate and mate preference was predicted to be positive. Ecologically and evolutionarily, however, one might expect that females avoid the costs of males that are at greater risk from frog-biting midges and midge-vectored pathogens (e.g. trypanosomes) (Bernal and Pinto, 2016) or increased predation risk to other enemies that may cue in on conspicuous ripples.

It seems likely that females can discriminate between call- and swat-induced ripples, as this would explain why increased call rates, with associated increases in call-induced ripples, are more attractive to females, while increased swat rates are less attractive. It is also possible that instead of affecting female preference directly, alternating high rates of call and swat ripples overloads female sensory processing (Hebets and Papaj, 2005). Such a cognitive effect may have been apparent in a female's latency to choose a male, though we found no impact of call or swat characteristics on latency. In a recent study, rival male signalers reduced their call rate in response to a male that frequently alternated between producing call and swat ripples (Leavell et al., 2023). It is intriguing to consider that the mechanism that causes male rivals to back down in a competitive interaction may have parallels in female mating decisions. We indeed found that higher swat rates limit male attractiveness to females as males increase their call rate, via an interactive effect. Thus, female preference appears to impose on males a trade-off between attracting mates and avoiding attacks.

In contrast to females, bats did not change preferences in response to swat ripples. Only call rate affected bat prey selection. That is, frog-biting midges can only influence bat foraging decisions by indirectly altering a male signaler's call rate, which occurs when frogs stop calling to swat attacking midges (Leavell et al., 2022). Our experimental design did not allow us to test the impact of call-induced ripples apart from the airborne call component. As such, we cannot tease apart whether the addition of call-induced ripples may have amplified the bats' preference for greater call rates or whether the airborne component alone impacted preference. Bats seem to prefer frog calls when call-induced ripples are present (Halfwerk et al., 2014), suggesting they are capable of detecting these and the more intense swat-induced ripples. Ultimately, considering that swat-induced ripples did not affect preference, we found no direct evidence that bats used ripple information in this study.

While bats in a simple foraging task as in our experiment might not depend on multi-sensory streams of information, it is possible that outside the lab, in more realistic heterogeneous environments where bats navigate through the complex forest while identifying and discriminating among prey, bats may rely more on actively probing the space via echolocation and may therefore make use of potentially redundant pieces of sensory information such as call- and swat-induced ripples. In such circumstances, bats may exhibit improved localizability of frog prey in the presence of swat ripples – in a parallel way to how calls with chucks increase the ability of bats to localize frogs calling in complex environments (Page and Ryan, 2008). Alternatively, when bat echolocation is unobstructed by acoustic clutter, a stream of bat calls reflecting off a swatting frog's body may be more salient to the bats than those echoing off ripples.

Emergent impacts of multiple eavesdroppers on the evolution of mating systems are potentially non-trivial, as mating signals can be important contributors to behavioral reproductive isolation and speciation (Ritchie, 2007). As mentioned previously, theory predicts that defenses that target one type of enemy likely increase a prey's risk of attack from other enemies in the community (Matsuda et al., 1996). We, however, do not find support for this prediction. While prey may compensate for additional risk by modifying other behaviors (Sih et al., 1998), male frogs do not appear to do so through changes to their signals. Such compensation would have appeared as an interaction between swat rate and one or more call characteristics, which was not seen in our bat model.

It was a surprise to find that changes in the ornamental chucks that frogs append to their calls had no direct influence on preference in either females or bats. The number of chucks that a male adds is known to affect both female mate choice and bat foraging behavior (Tuttle and Ryan, 1981; Page and Ryan, 2008; Ryan et al., 2019). In one study, the proportion of chucks between two calling males explained 84% of the variation in call preference among females and 74% of the variation in bat preference (Akre et al., 2011). We did not find this effect here. The difference between our findings and those from other studies may be explained by the respective experimental designs, as previous studies have generally tested female túngara frogs and bats in controlled conditions that prevented frog-biting midges from influencing interactions. This communication network, made up of male and female frogs, predatory bats and midges (and other eavesdropping predators; Bernal and Page, 2023), is clearly a dynamic, integrated system that fluidly responds to multisensory streams of signals and cues. Previous studies examining slices of this communication network, such as solely the effect of airborne call components on females or bats, have revealed the strong salience and influence of male chucks. Here, by accounting for interactive effects among target and non-target receivers in the system and featuring more of the possible sensory information available to receivers, we found that the drivers of receiver behavior are complex and context dependent. As mentioned previously, we did not find direct evidence that ripples influenced bat behavior, yet we cannot discount the possibility that the incorporation of ripples was enough to alter female and bat responses to the relative numbers of chucks among competing males. Receivers sense airborne chuck components before the slower propagating ripples. Females may thus initially prefer the airborne component of a male that is chucking more often, but her preference may then be reduced once she senses the call ripples. Bats may also show an initial preference for greater chucks, but once approaching two calling frogs, may attend to call ripples and instead choose the male calling more frequently. Many sensory and cognitive mechanisms might explain this effect. The call ripples may, for example, improve localizability, distract receivers from their initial preferences or simply be more stimulating.

We did find, however, that males with less complex calls were more likely to attract females if they swatted less frequently. This effect surprisingly appears to make males with low-complexity calls, if they swat infrequently, more attractive than the average calling male. Such a finding suggests that swat ripples are not inherently aversive to females but are only attractive in strict moderation. Infrequent swatting may be an honest signal of a male's capacity to deal with midge attacks and the associated loss of blood and increased pathogen load, leading to selection favoring female preference for infrequent ripples. A non-mutually exclusive physiological explanation could be that infrequent ripples do not overload a female's sensory system or reduce habituation to them. Additionally, frequent high-intensity ripples may be difficult for females to discriminate from other sources of ripple cues, such as wading predators, leading to avoidance, whereas fewer swat ripples are more likely to be associated with a signaling male.

Regardless of the mechanisms, we found that micropredator attacks via their swat-induced ripples may sometimes make conventionally less attractive males (producing low-complexity calls) more attractive to females, and more attractive males (producing high call rates) less attractive to females. In this way, micropredators may temper the relative attractiveness of males at a chorus. These findings extend previous work showing that micropredators dynamically limit a male's call rate and complexity (Leavell et al., 2022) by revealing how anti-eavesdropper defense strategies can also elicit context-dependent female preferences. Overall, by altering the influence of signal components on female mating and bat foraging decisions, these and other eavesdropping micropredators may play an important yet previously unknown role in the evolution of signals and communication systems.

In summary, eavesdropping micropredators shape the mating decisions of a signaler's target receivers (potential female mates) and foraging decisions of non-target receivers (eavesdropping predators). This effect occurs indirectly via the swatting defense the signaler uses to combat eavesdropping micropredator attacks. We found that the specific mechanism (via changes to call rate or water-borne ripples) is context and receiver specific. Though different receivers in this communication network overlap in their perception of signals, the asymmetric effects of anti-midge swatting defenses are not entirely unexpected given such disparate ecologies and sensory systems as those of frogs and bats. It remains unclear to what extent eavesdropping micropredators, or eavesdropping natural enemies in general, have similar effects in other communication networks. Intriguingly, the dynamic ecological effects of micropredators on signal and defensive variation in túngara frogs are similar in their complexity to the outcomes of selection by eavesdropping parasitoids on the varied signaling strategies in Hawaiian populations of field crickets (Gallagher et al., 2024). What is clear, however, is that to understand selection on signals and how communication systems evolve, a full account of direct and indirect interactions across multi-receiver networks is needed.

## Supplementary Material

10.1242/jexbio.250360_sup1 Supplementary information
