# Supplementary material for: Variation in sexual signals and defensive strategies elicits receiver-dependent shifts in attractiveness
Source: J Exp Biol. 2025 Jul 25;228(15):jeb250360. doi: 10.1242/jeb.250360 (PMC12319409; doi:10.1242/jeb.250360)
Supplement: Supplementary information [file jexbio-228-250360-s1.pdf]

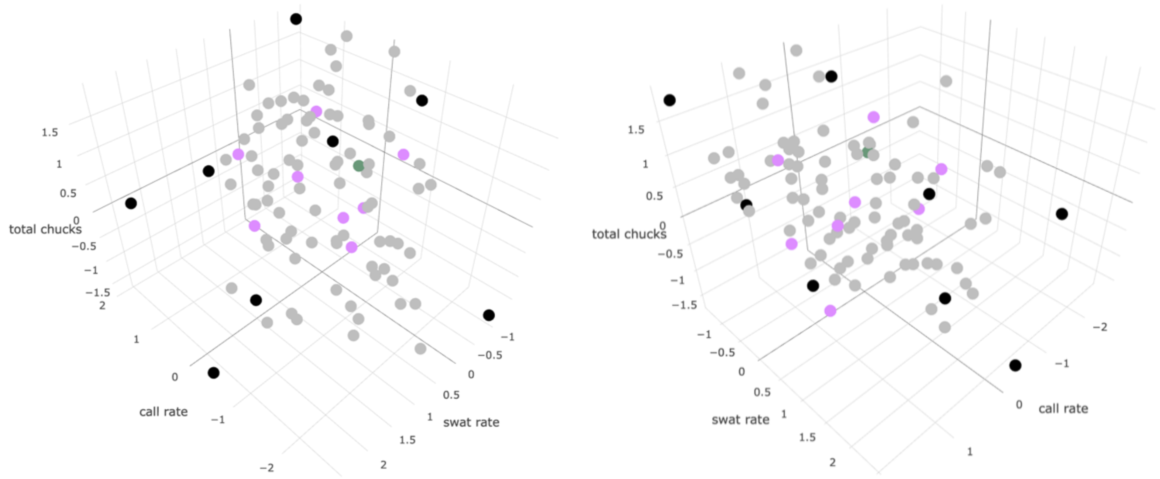

Fig. S1. Three-dimensional space of standardized and scaled call characteristics and anti-midge swatting defense strategies from individual male túngara frogs. Each dot represents one male recorded *in situ*. Black dots represent extreme samples, pink dots represent samples approximately 1 standard deviation from the mean, and the green dot represents the sample closest to the mean. The gray dots are samples of males not used in experiments, but used to determine z-scores.

Journal of Experimental Biology • Supplementary information

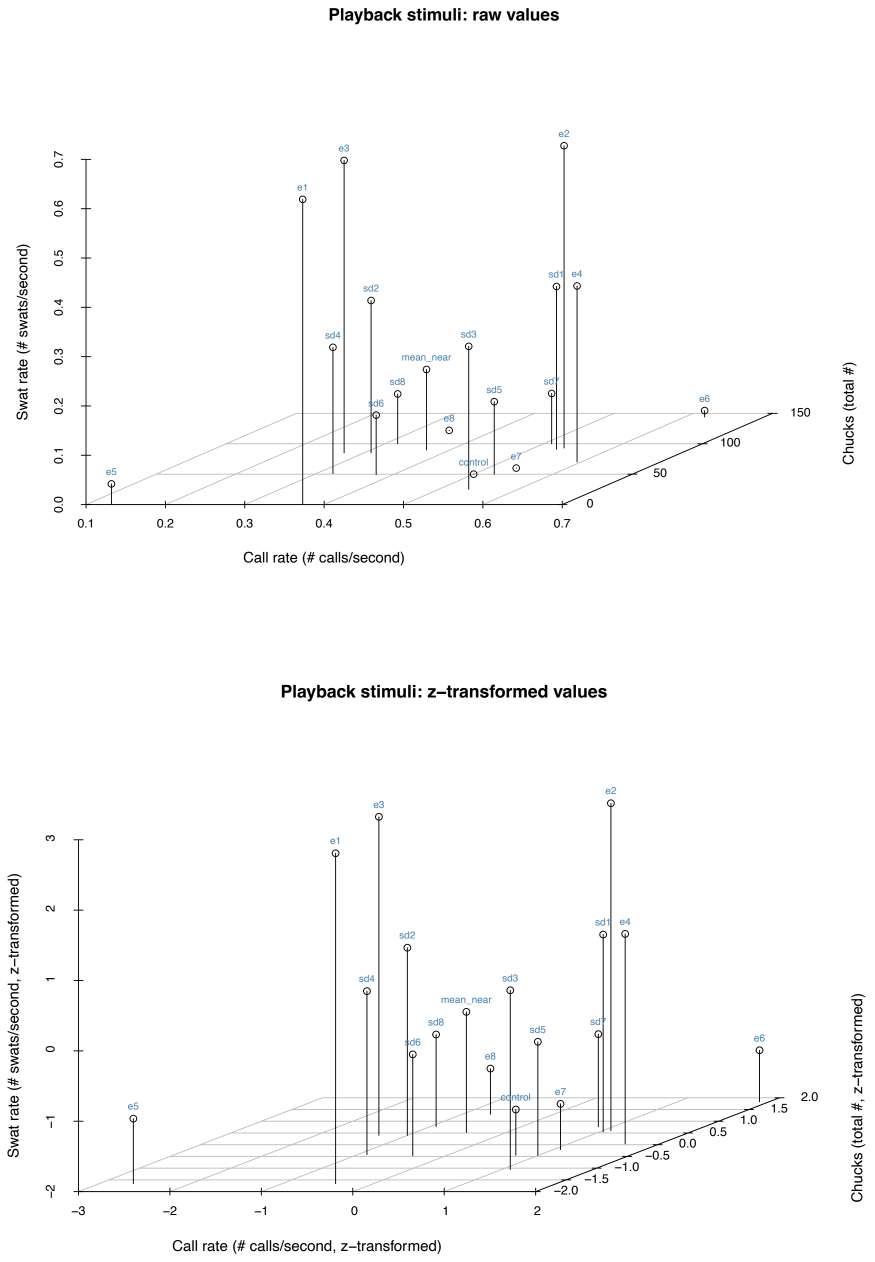

Fig. S2. Playback stimuli. (Top panel) Raw values of playback stimuli, shown in three-dimensional space for call characteristics and anti-midge swatting defense strategies from individual male túngara frogs. "e" indicates extreme samples, "sd" indicates samples approximately 1 standard deviation from the mean, "mean_near" indicates the sample closest to the mean, and "control" indicates the stimulus played back against treatment stimuli in trials. (Bottom panel) The same values as top panel, but z-transformed as in Figure S1.

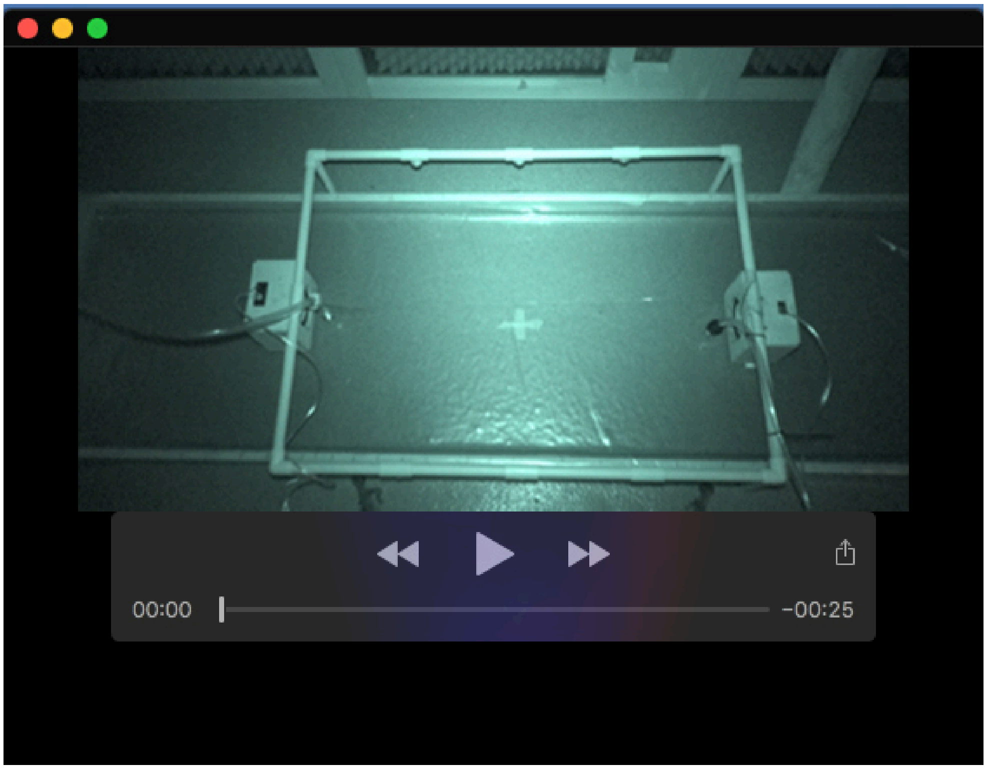

Movie 1. Example of a trial from the female choice experiment.

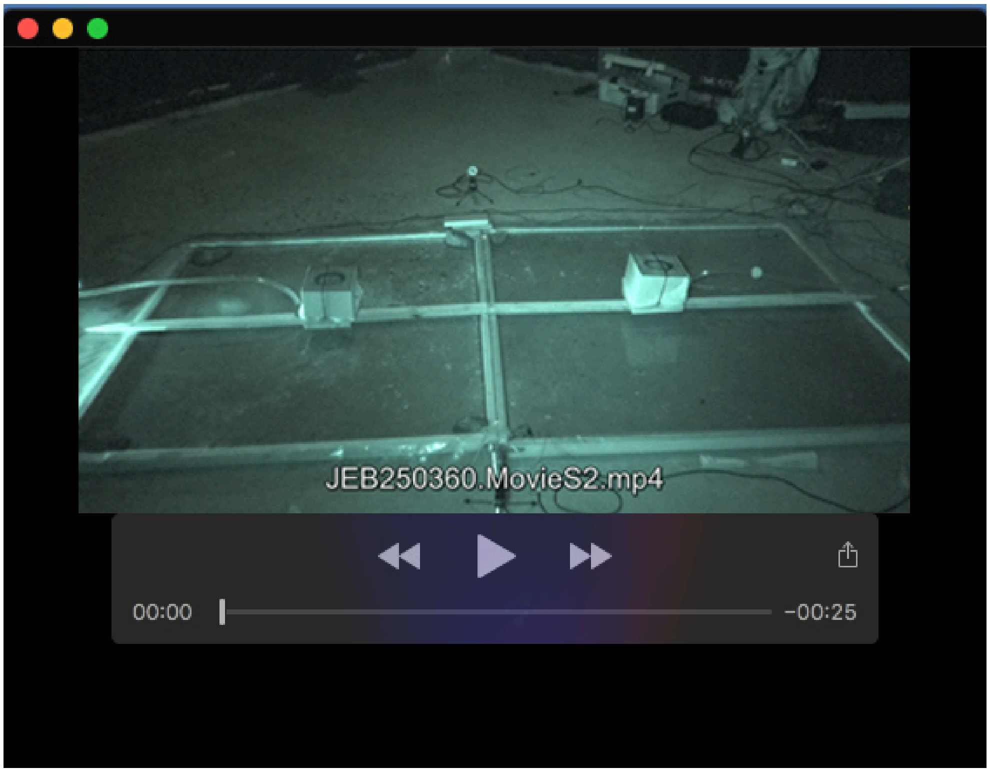

Movie 2. Example of a trial from the bat foraging experiment.

Journal of Experimental Biology • Supplementary information

Supplementary Materials and Methods

Procedure for Video Analysis of Female Frog Behavior

We determined a choice was made when:

1. The frog slowed down or stopped within approximately 10 cm of the front or back of the speaker.
2. If moving without slowing within 1 body length of the ripple nozzle. Males in the wild will sometimes grasp moving females that come within this distance of a male (Leavell, pers. obs.).

We assessed a total of 216 trials. During the experiment there were trials in which a choice was not immediately obvious, in which case we performed a second "redo" trial. However, if upon video review we determined a choice had been made during the first trial, we included this trial and omitted the result of the second "redo" trial in the analysis. We also omitted trials in which the female committed a "foul", defined as when time expired or the female jumped out of the arena without making a choice. Additionally, one trial was removed due to interference by external conditions.

Journal of Experimental Biology • Supplementary information
